# Heterotrimeric G protein-dependent WNT-5A signaling to ERK1/2 mediates distinct aspects of microglia proinflammatory transformation

**DOI:** 10.1186/1742-2094-9-111

**Published:** 2012-05-30

**Authors:** Carina Halleskog, Jacomijn Petronella Dijksterhuis, Michaela Brita Christina Kilander, Javier Becerril-Ortega, Juan Carlos Villaescusa, Eva Lindgren, Ernest Arenas, Gunnar Schulte

**Affiliations:** 1 Dept. Physiology & Pharmacology, Sec. Receptor Biology & Signaling, Karolinska Institutet, Nanna Svartz väg 2, Stockholm, S-17177, Sweden; 2 Dept. Medical Biochemistry & Biophysics, Sec. Molecular Neurobiology, Karolinska Institutet, Retzius väg 8, Stockholm, S-17177, Sweden

**Keywords:** Frizzled, Heterotrimeric G proteins, MAPK, Non-canonical WNT signaling, Cyclic AMP, Microglia, Neuroinflammation

## Abstract

****Background**:**

WNT-5A signaling in the central nervous system is important for morphogenesis, neurogenesis and establishment of functional connectivity; the source of WNT-5A and its importance for cellular communication in the adult brain, however, are mainly unknown. We have previously investigated the inflammatory effects of WNT/β-catenin signaling in microglia in Alzheimer's disease. WNT-5A, however, generally recruits β-catenin-independent signaling. Thus, we aim here to characterize the role of WNT-5A and downstream signaling pathways for the inflammatory transformation of the brain's macrophages, the microglia.

****Methods**:**

Mouse brain sections were used for immunohistochemistry. Primary isolated microglia and astrocytes were employed to characterize the WNT-induced inflammatory transformation and underlying intracellular signaling pathways by immunoblotting, quantitative mRNA analysis, proliferation and invasion assays. Further, measurements of G protein activation by [γ-^35^ S]GTP binding, examination of calcium fluxes and cyclic AMP production were used to define intracellular signaling pathways.

****Results**:**

Astrocytes in the adult mouse brain express high levels of WNT-5A, which could serve as a novel astroglia-microglia communication pathway. The WNT-5A-induced proinflammatory microglia response is characterized by increased expression of inducible nitric oxide synthase, cyclooxygenase-2, cytokines, chemokines, enhanced invasive capacity and proliferation. Mapping of intracellular transduction pathways reveals that WNT-5A activates heterotrimeric G_i/o_ proteins to reduce cyclic AMP levels and to activate a G_i/o_ protein/phospholipase C/calcium-dependent protein kinase/extracellular signal-regulated kinase 1/2 (ERK1/2) axis. We show further that WNT-5A-induced ERK1/2 signaling is responsible for distinct aspects of the proinflammatory transformation, such as matrix metalloprotease 9/13 expression, invasion and proliferation.

****Conclusions**:**

Thus, WNT-5A-induced and G protein-dependent signaling to ERK1/2 is important for the regulation of proinflammatory responses in mouse primary microglia cells. We show for the first time that WNT-5A/G protein signaling mediates physiologically important processes in primary mammalian cells with natural receptor and G protein stochiometry. Consequently, WNT-5A emerges as an important means of astrocyte-microglia communication and we, therefore, suggest WNT-5A as a new player in neuroinflammatory conditions, such as neurodegenerative disease, hypoxia, stroke, injury and infection.

## **Background**

WNT-5A belongs to the WNT family of secreted lipoglycoproteins which is composed of 19 WNTs in mammals and is crucial for stem cell proliferation and differentiation during embryonic development [1-3]. During central nervous system (CNS) development, WNTs regulate stem cell proliferation and differentiation, functional integration, axonal growth, neuronal connectivity and activity [4,5]. Recent evidence also indicates that WNTs in the adult CNS regulate stem cells and maintain tissue homeostasis, and they have also been implicated in neurological disorders [5,6].

WNTs interact with and activate several different cell surface receptors of which the Class Frizzled (FZD) belongs to the group of G protein-coupled receptors (GPCRs) [7]. WNT-5A, in contrast to, for example, WNT-1 and -3A, generally activates β-catenin-independent signaling [3,8-10]. In various cell systems it was shown that WNT-5A activates WNT/Ca^2+^ signaling, the WNT/RAC1 and WNT/RHO pathway [11] involving the phosphoproteins disheveled 1, 2, 3 (DVL1, 2, 3), which are viewed as a signaling hub relaying many WNT signaling routes [12]. Further, heterotrimeric G proteins have been implicated in WNT/FZD signal transduction [13-16] and, indeed, recent studies confirmed a previous supposition that WNTs can induce the activation of heterotrimeric G proteins [17-19]. Among classical downstream targets of GPCR signaling are the mitogen-activated protein kinases (MAPK) and especially the extracellular signal-regulated kinases ERK1/2 [20], which can be activated by many GPCRs irrespective of which G protein family the receptors associate with [21,22].

Here, we examined the WNT-5A-induced communication to ERK1/2 and its relevance for the inflammatory transformation of mouse primary microglia, the immunocompetent cells of the CNS [23]. We show that WNT-5A is expressed in astrocytes and that it induces a rapid and transient phosphorylation of the ERK1/2 by recruitment of Gα_i/o_ heterotrimeric G proteins, phospholipase C (PLC), calcium-dependent protein kinase (PKC) and the MAPK/ERK1/2 kinase (MEK1/2) in microglia. Further, recombinant and purified WNT-5A induces a proinflammatory transformation of mouse primary microglia as determined by increased expression of inflammatory markers, cytokines, chemokines and matrix metalloproteases, enhanced invasive capacities, and increased microglial proliferation. Employing the MEK1/2 inhibitor SL327 [24], we found that microglial proliferation, invasion and expression of matrix metalloproteases depend on the activation of heterotrimeric G proteins and the subsequent phosphorylation of ERK1/2. Thereby, we identify a novel function of WNT-5A as a proinflammatory regulator of microglia and pinpoint a novel signal transduction pathway downstream of WNT-5A with the Gα_i/o_ protein-dependent activation of ERK1/2 underlying distinct aspects of the proinflammatory transformation of microglia.

## **Methods**

### **Immunohistochemistry**

Adult C57Bl6 mouse brains were collected and fixed in 4% paraformaldehyde (PFA) in PBS (4% PFA) for 24 h at 4°C. Fixed brains were cryoprotected in 15% to 30% sucrose in PBS, and sectioned using a Microm HM 560 cryostat. Mouse brains were sectioned at 16 μm thickness and mounted on Superfrost Plus glass slides (Thermo Fisher Scientific Inc. Rockford, IL, USA). Sections were treated for antigen retrieval (#S1699, DAKO Sweden AB, Stockholm, Sweden), washed with PT20 (PBS, 0.5% Tween-20), blocked with PBTA (PBS, 0.1% BSA, 0.3% Triton X-100, 5% Donkey serum) and incubated with primary antibodies diluted in PBTA, overnight at 4°C. The primary antibodies in this study included the following: rat anti-WNT-5A (1:50, #MAB645, R&D Systems, Minneapolis, MN, USA), rabbit anti-IBA1 (1:500, #019-19741, Wako, Neuss, Germany) and mouse anti-glial fibrillary acidic protein (GFAP) Cy3 conjugated (1:250, #C9205, Sigma-Aldrich Sweden AB, Stockholm, Sweden). After washing with PT20, slides were incubated with secondary antibodies diluted in PBTA for one hour at room temperature (Alexa anti-rat 488 and Alexa anti-rabbit 647, Invitrogen, Stockholm, Sweden). Slides were washed and mounted with mounting medium (#S3023, DAKO). Images were captured using a confocal microscope (LSM5 Exciter, Zeiss, Jena, Germany), and a maximum intensity projection of a Z-stack is presented (5 μm).

### **Primary cell cultures**

**Figure 1:**
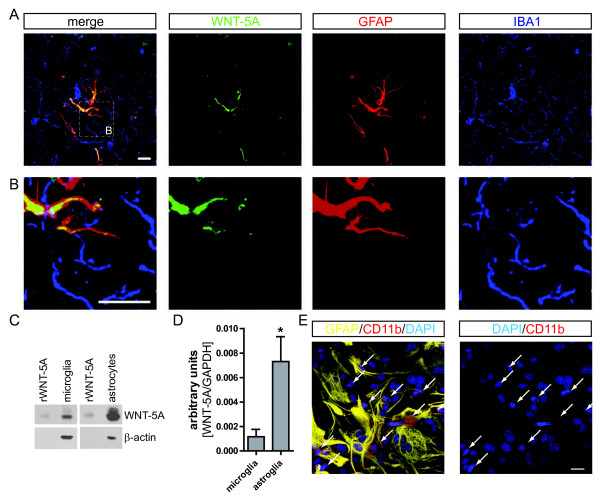
**WNT-5A expression in mouse GFAP**^**+**^**astrocytes.** (**A**) Immunohistochemistry was performed on adult mouse brain sections using an anti-WNT-5A antibody in combination with anti-glial fibrillary acidic protein (GFAP) and ionized calcium-binding adaptor protein1 (IBA1) antibodies as astrocyte and microglia marker, respectively. Merge presents the overlay of IBA1, GFAP, WNT-5A. Size bar −2 μm. The images represent a maximum intensity projection of a Z-stack of 5 μm thickness. The white square marked ‘B’ indicates the area magnified in B: (**B**) Close up of a GFAP^+^ astrocyte reveals the expression of WNT-5A in this cell type. (**C**) shows immunoblot detection of recombinant WNT-5A (rWNT-5A; 375 ng/lane) in comparison to lysates from mouse primary microglia and mixed astrocyte cultures. β-actin serves as a loading control. (**D**) The bar graph depicts expression levels of WNT-5A mRNA in mouse primary microglia and mixed astrocyte cultures measured by QPCR. Data are normalized to GAPDH expression and analyzed with a non-parametric Mann–Whitney test. *, *P* < 0.05; **, *P* < 0.01; ***, *P* < 0.001. n = 4 to 8. (**E**) shows indirect immunocytochemistry of mixed astrocyte cultures employing anti-GFAP as astrocyte and anti-CD11b as microglia markers. DAPI is used as nuclear counterstain. Image represents a maximum image projection of an 8 μm Z-stack. Size bar 20 μm. The frame shows 63 cells in total and 10 CD11b-positive microglia (arrows). Routinely 10% to 18% microglia were observed (n = 4). DAPI, 4',6-diamidino-2-phenylindole; GADPH, glyceraldehyde 3-phosphate dehydrogenase; n, number.

Microglia cells and astrocytes were prepared from newborn C57Bl 6 mice (postnatal day 1 to 3) according to the ethical permits N144/08 and N436/10; local ethical committee Stockholms Norra Djurförsöksetiska Nämnd. After decapitation, while the brains were kept in ice cold Hanks Balanced Salt Solution (HBSS; Invitrogen) blood vessels and meninges were removed with sterile forceps. Brains were rinsed four times with HBSS, and homogenized in (D)MEM, penicillin (50 U/mL), streptomycin (50 μg/mL), L-glutamine (2 mM), 10% fetal bovine serum (FBS) and Fungizon (0.5 μg/ml) (all from Invitrogen), with a 10 ml pipette, then ten times with a 10 ml syringe and needle (23 G) and then filtered through a cell strainer (70 μm; BD Falcon, Stockholm, Sweden) into a 50 ml falcon tube. After ten minutes centrifugation (900 g) the pellet was resuspended in fresh medium, filtered again and cultured in 75 cm^2^ flasks (one brain/flask). Medium was changed every fourth day, and after 10 to 12 days microglia cells were separated from the underlying astrocytic monolayer by gentle agitation. The composition of primary mixed astrocyte cultures used for Figure 1 was examined by immunocytochemistry using anti-GFAP (astrocyte marker) and anti-CD11b (microglia) revealing a low degree (10% to 18%) of contamination with CD11b-positive microglia (Figure 1E) comparable to what was reported earlier [25]. Microglia cells could be harvested three times from the same batch. The purity of the microglia cultures (>95%) was validated by cytochemistry using fluorescein isothiocyanate (FITC)-conjugated Griffonia simplicifolia isolectin B4 (Sigma-Aldrich Sweden AB, Stockholm, Sweden) or anti-CD11b antibody in combination with anti-GFAP staining to assess contamination with astrocytes [see Additional file 1: Figure S4]. Cells were challenged 24 h after plating. For isolation of primary mixed astrocyte cultures, the remaining cells were trypsinated after microglia harvest and seeded according to experimental requirements.

### **Immunoblotting**

Microglia cells were plated in a 24- or 48-well plate at a density of 150,000 or 100,000 cells/well, respectively. Twenty-four hours later, medium was changed to starvation medium (MEM, penicillin (50 U/mL), streptomycin (50 μg/mL; all from Invitrogen) overnight. Microglia cells were stimulated with recombinant carrier-free WNT-5A (CF WNT-5A; R&D Systems) and inhibitors were added 30 min or 24 h (pertussis toxin (PTX) before stimulation. Stimulation was stopped by addition of lysis buffer (10% glycerol, 1% SDS, 100 mM Tris/HCl pH 7.4, bromophenol blue, 1% mercaptoethanol). Lysates of mixed astrocyte cultures were prepared after cells were cultured for 24 hours in 24 well plates (500,000 cells/well). Lysates were homogenized with a hamilton syringe and samples were then separated through an 8% or 10% SDS PAGE and electrotransferred to Immobilon-P membranes (Millipore, Solna, Sweden). After blocking with 3% milk in TBS-T, the membranes were incubated with primary antibodies in blocking buffer: rabbit anti-phosphospecific Thr202/Tyr204 ERK1/2 (1:1000; #9101, Cell Signaling Technology, Boston, MA, USA), rabbit anti-phosphospecific MEK1/2 (1:250; #9121, Cell Signaling Technology), rabbit anti-P-LRP6 (1:1000; #2568, Cell Signaling Technology), mouse anti-β-actin (1: 30.000; #A5441, Sigma), mouse anti-DVL3 (1:500; #sc-8027, Santa Cruz Biotechnology, Santa Cruz, CA, USA), mouse anti-β-catenin (1:1000; #610154, BD Bioscience, Stockholm, Sweden), rat anti-WNT-5A (1:500; #MAB645, R&D Systems), rabbit anti-iNOS (1:1000; #N7782, Sigma), goat anti-COX-2 (1:500; #sc-13474, Santa Cruz) and goat anti-TNFα (1:500; #AF-410-NA, R&D Systems) overnight at 4°C. The proteins were immunodetected with appropriate horseradish peroxidase-conjugated secondary antibodies (goat anti-rabbit and goat anti-mouse (Pierce, Rockford, IL, USA) and rabbit anti-goat (Sigma) that were visualized by the enhanced chemiluminescence method (Western-Lightning, PerkinElmer, Waltham, MA, USA).

### **RNA isolation and PCR**

Microglia cells and mixed astrocytes cultures were seeded in six-well plates (Costar) at a density of 1.2 million/well. After 24 hours, cells were starved for four hours and then stimulated for six hours with CF WNT-5A/control (ctrl). RNA was isolated using the RNeasy Mini kit (Qiagen, Hilden, Germany) and transcribed to cDNA using the high-capacity cDNA Archive kit (Applied Biosystems, Foster City, CA, USA). QPCR was performed in triplicate on an ABI Prism 7000 sequence detector and with the Taqman gene expression assay (Applied Biosystems) according to the manufacturer’s instructions. Primer efficiency was previously tested by the manufacturer and shown to be close to 100% for all used primers. Additionally, primer efficiency was evaluated using the C_T_ slope method over six serial cDNA dilutions indicating similar efficiencies close to 100% for all primers at the conditions used for analysis. All primer pairs were from Applied Biosystems: TNF-α: Mm00443260_g1, IL1β: Mm00434228_m1, IL6: Mm99999064_m1, IL12: Mm01288991_g1, CCl12: Mm01617100_m1, CCl7: Mm00443113_m1, MMP9: Mm004429941_m1, MMP13: Mm00439491_m1, CD69: Mm01183378_m1, CD40: Mm00441891_m1, Cox-2 (Ptgs2): Mm00478374_m1, Fzd1: Mm01320682_s1, Fzd2: Mm02524776_s1, Fzd3: Mm00445923_m1, Fzd4: Mm03053556_s1, Fzd5: Mm00445623_s1, Fzd6: Mm00433383_m1, Fzd7: Mm01255614_s1, Fzd8: Mm00433419_s1, Fzd9: Mm01206511_s1, Fzd10: Mm00558396_s1. As an internal reference standard primers for GAPDH were used (ΔC_t_ = C_t, target gene_ - C_t, GAPDH_), ΔC_t_ was presented as relative fold change in gene expression (mean ± standard error of the mean (s.e.m)) indicated as arbitrary units, target gene/GAPDH. RT-PCR was performed with cDNA from unstimulated microglia in a 2720 Thermo Cycler (Applied Biosystems) using the following standard program: five minutes at 94°C; 30 cycles of 30 seconds at 94°C; 45 seconds at T_anneal_; one minute at 72°C; ten minutes at 72°C. PCR products were run on 2.5% agarose gels and visualized using GelRed fluorescent nucleic acid gel stain (Biotium, Stockholm, Sweden). For primer sequences and T_anneal_ see [17].

### **[γ**^**35**^**S]-GTP assay in microglia membranes**

Cells were seeded in 60 mm dishes (4 to 5 million cells/dish). After 48 hours, cell plasma membrane proteins were isolated using the ProteoJet membrane protein extraction kit (Fermentas, Heidelberg, Germany) according to instructions provided by the manufacturer. [γ^35^ S]GTP assay was performed as previously described [17]. In brief, the assay was performed in a 96-well filter plate (Millipore) in which 10 μg/well of pre-activated (one hour, 37°C) membrane protein were incubated (one hour, 30°C) with 300 ng/ml WNT-5A or vehicle control (0.01% BSA in PBS) in the presence of 0.25 nM [γ^35^ S]-GTP (Perkin Elmer) and 5 μM GDP (Sigma-Aldrich). All substances and protein were diluted in assay buffer (100 mM NaCl, 10 mM MgCl_2_, 20 mM HEPES, pH 7.4) to a final volume of 300 μl/well. Subsequently, the plate was washed three times with 0.9% saline solution using vacuum filtration and dried overnight at 60°C. Thirty microliters per well Optiphase supermix scintillation liquid (Perkin Elmer) were added and the incorporation of [γ^35^ S]-GTP was measured using a MicroBeta^2^ LumiJET Scintillation counter (Perkin Elmer).

### **cAMP assay**

Intracellular cAMP levels were determined using a competitive protein binding assay and ^3^ H]-cAMP. Briefly, cells were plated into 24 well microplates, grown overnight and stimulated at 37 °C. The reaction was stopped by addition of perchloric acid to a final concentration of 0.4 M and incubation on ice. After lysis and neutralization with KOH in 50 mM Tris, the supernatant was examined for cAMP content as described elsewhere [26].

### **[Ca**^**2+**^**]**_**i**_**imaging**

Primary microglia cells were harvested and placed in 35 mm dishes (100,000 cells per dish) coated with ECM Gel from Engelbreth-Holm-Swarm murine sarcoma (Sigma-Aldrich). Cells were loaded for 15 minutes at 37°C with 10 μM Fluo-3/AM and 0.2% pluronic acid (F-127, Invitrogen) and incubated for an additional 15 minutes at 37°C in (D)MEM with HEPES (12 mM) at pH 7.45. Experiments were performed at 37°C with continuous perfusion at 1 ml/minute with a peristaltic pump, on the stage of a Zeiss inverted microscope and water immersion Zeiss C-Apochromat 40x/1.20 water correction M27 objective. Fluo-3 (excitation: 488 nm, emission: 526 nm; recorded range 503–629 nm) images were acquired every four seconds using Zen 2009 software (Zeiss). Measurements were performed on microglial cell bodies by using amplification rates that prevent saturation of the fluorescence signal and a preliminary calibration of the Ca^2+^ measurements.

### **Mesoscale**

Primary microglia were stimulated with WNT-5A for 24 hours. The cell medium was cleared by centrifugation and analyzed for TNFα protein by a 96 well mouse cytokine immunoassay (Meso Scale Discovery, Gaithersburg, MD, USA) according to the manufacturer’s instructions. TNFα concentrations in samples were compared with a dilution standard curve of recombinant TNFα. Plates were analyzed with the SECTOR instrument and software.

### **MTT-assay**

Cells were seeded in a 96-well plate (40,000 cells/well). The next day, medium was changed to starvation medium (total volume 100 μl/well) for an additional 24 hours, after which cells were challenged with 300 ng/ml CF WNT-5A (R&D Systems). After 24 hours of stimulation, 20 μl of 3-(4,5-dimethylthiazol-2-yl)-2,5-diphenyltetrazolium bromide (MTT) reagent solution (5 mg/ml in PBS; Sigma) were added to each well, and also in a set of wells without cells to analyze background, and incubated for 3.5 hours at 37°C to allow for mitochondrial reduction of tetrazoles to formazan. After aspiration of the MTT solution, 150 μl/well solvent (4 mM HCl and 0.1% Nonidet P-40 in isopropanol) were added and the plate was agitated for 20 minutes on an orbital shaker. Absorbance was read at 570 nm using a plate reader with a reference filter at 620 nm. Increases in MTT were interpreted as increased proliferation since mitochondrial/metabolic activity correlates with cell number.

### **Invasion assay**

A collagen mixture containing 10x (D)MEM, 5% NaHCO_3_ (pH 8.97), HEPES 1 M (pH 7.5) and rat collagen I (final concentration 3.6 mg/ml, Gibco, Stockholm, Sweden) was placed on top of an extracellular matrix (ECM) gel (Sigma) coated 24-well glass bottom plate (MatTek Corporation, Ashland, MA, USA). After 90 minutes, the gel was fully polymerized and 50,000 fluorescently labeled primary microglia (cell tracker red 5 μM for 30 minutes, Invitrogen) in αMEM growth medium containing either CF WNT-5A (300 ng/ml; R&D systems) or PBS were placed on top of the layer. The next day, invasion of microglia into the collagen matrix was measured with a confocal microscope LSM710 system (Zeiss), scanning three random positions per condition and using Z-stack measurement with an EC Plan-Neofluar 10x/0.30 M27 objective, a 561 nm laser for excitation and an emission range of 566/683 nm. During scanning, cells were kept in a humidified atmosphere at 37°C and 5% CO_2_. Z-stacks were acquired with ZEN 2009 software. To quantify the percentage of invasion (cells that moved a minimum of 20 μm from the top layer), the invading cells were divided by the total number of cells, employing the particle tracker function in Imaris 7.2 software (Bitplane). The results are shown for three independent experiments and invasion is normalized to the control.

### **Statistical analysis**

Statistical and graphical analysis was performed using the Graph Pad Prism 5 software. All data, except quantitative PCR experiments were analyzed with one-way analysis of variance (ANOVA) followed by a Bonferroni's multiple comparison post hoc test. Quantitative PCR experiments were analyzed with a non-parametric Mann–Whitney test. All experiments were repeated at least three independent times. Levels of statistical significance: *, *P* < 0.05; **, P < 0.01; ***, *P* < 0.001.

## **Results**

### **WNT-5A is expressed in astrocytes in vivo**

In order to address the role of WNT-5A for intercellular communication in the adult brain, we asked first of all, which cells express the WNT-5A protein. Immunohistochemistry in brain sections from adult mice with a rat-anti WNT-5A antibody in combination with an astrocyte (anti-GFAP) and a microglia marker (anti-IBA1) revealed strong overlap of WNT-5A- and GFAP-like immunoreactivity in the whole brain. Figure 1A, B show cortical WNT-5A- and GFAP-positive astrocytes and closely associated with IBA1-positive but WNT-5A-negative microglia. These data are supported by detection of WNT-5A protein and mRNA in primary microglia and mixed astrocyte cultures with immunoblotting (Figure 1C) and quantitative PCR (Figure 1D), respectively. The high expression levels of WNT-5A in mixed astrocyte cultures compared to microglia suggest a possible paracrine WNT-5A-based communication from astrocytes to microglia.

### **WNT-5A stimulation of microglia results in phosphorylation of ERK1/2**

**Figure 2:**
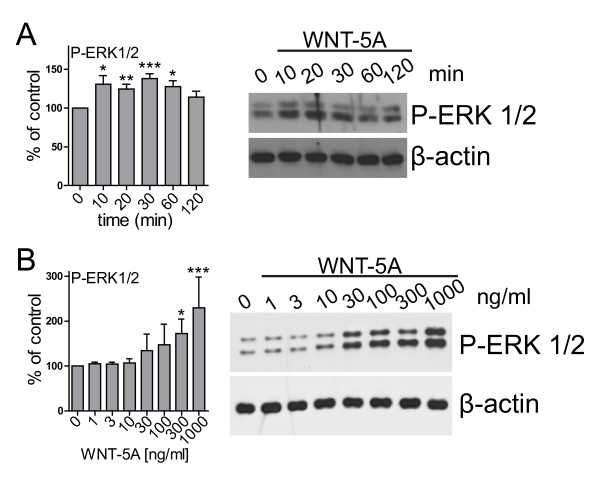
**WNT-5A induced a transient phosphorylation of ERK1/2.** WNT-5A stimulation of mouse primary microglia induced ERK1/2 phosphorylation in a time- (**A**; 300 ng/ml WNT-5A) and dose-dependent (**B**; 30 minutes WNT-5A) manner. Bar graphs provide a summary of densitometric analysis of three independent experiments. Data were normalized to the response in ctrl-stimulated microglia. Variation is shown as s.e.m. (n ≥ 3). *, *P* < 0.05; **, *P* < 0.01; ***, *P* < 0.001. ctrl, control; ERK1/2, extracellular signal-regulated kinase 1/2; n, number; s.e.m, standard error of the mean.

Activation of MAPKs is of central importance for the regulation of microglia activity in response to a plethora of stimuli ranging from lipopolysaccharides to cytokines, chemokines and neurotransmittors [27-29]. We hypothesized that WNT-5A, which is expressed in and secreted from astrocytes rather than by microglia themselves (Figure 1A, B) could regulate the inflammatory potential of microglia, possibly through MAPKs. These cells express suitable WNT-5A surface receptors as shown in Additional file 1: Figure S1 and in a previously published study [30]. In mouse primary microglia isolated from newborn mice, recombinant and purified WNT-5A increased the phosphorylation of ERK1/2 in a time- and dose-dependent manner (Figure 2A, B). The WNT-5A-evoked ERK1/2 phosphorylation was rapid with increased levels at ten minutes post-stimulation and maximal induction (138.2 ± 6.2% of ctrl; mean ± s.e.m.) at 30 minutes. Two hours after WNT stimulation, P-ERK1/2 levels had returned to basal. Substantial efficacy of WNT-5A could be detected at 300 ng/ml, a dose which has been previously shown to be effective to induce WNT-5A signaling in other cell types [31,32]. In order to confirm the specificity of the WNT-5A-induced P-ERK1/2 response we employed recombinant soluble FZD-related protein 1, which completely blocked the WNT-5A-induced ERK1/2 phosphorylation [see Additional file 1: Figure S2].

In parallel to the WNT-5A-induced ERK1/2 phosphorylation, we assessed phosphorylation of LRP6, β-catenin stabilization and the formation of phosphorylated and shifted DVL3 (PS-DVL3) [see Additional file 1: Figure S3]. According to previous findings in other cell types, WNT-5A did not activate the WNT/β-catenin pathway, but increased the formation of PS-DVL in a time- and dose-dependent manner [8,31].

### **WNT-5A activates heterotrimeric Gα**_**i/o**_**proteins and induces [Ca**^**2+**^**]**_**i**_**in microglia**

**Figure 3:**
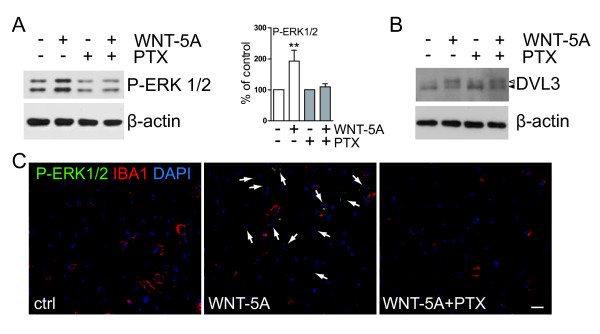
**WNT-5A-induced ERK1/2 phosphorylation requires activation of Gα**_**i/o**_**.** (**A**) Primary microglia, pretreated with PTX (100 ng/ml, overnight), were stimulated with 300 ng/ml WNT-5A for 30 minutes and their total cell lysate was analyzed for P-ERK1/2 by immunoblotting. β-actin serves as a loading control. Densitometric analysis of three independent experiments is summarized in the bar graph (error bars - s.e.m.). **, *P* < 0.01. (**B**) In a similar stimulation paradigm, cells were lysed after two hours and the lysate was analyzed for the formation of PS-DVL3 (filled triangle: DVL3, open triangle: PS-DVL3). n = 3. (**C**) Indirect immunocytochemistry and confocal microscopy were used to detect and localize P-ERK1/2 in ctrl, WNT-5A (300 ng/ml, 30 minutes) and WNT-5A/pertussis toxin (PTX, 100 ng/ml)-treated mouse primary microglia. IBA1 was used as a microglia marker and DAPI as nuclear counterstain. Arrows mark WNT-5A-responsive microglia with increased P-ERK1/2 levels (n = 3). Size bar = 50 μm. ctrl, control; DAPI, 4',6-diamidino-2-phenylindole; DVL, disheveled; ERK1/2, extracellular signal-regulated kinase 1/2; IBA1, ionized calcium-binding adaptor protein1; n, number; P-ERK1/2, ERK1/2 phosphorylation; PS-DVL, phosphorylated and shifted DVL; s.e.m., standard error of the mean.

Since WNT-5A-induced signaling to ERK1/2 has not been described in detail yet, we aimed to characterize more closely the underlying signaling components. First of all, we used PTX as an inhibitor of Gα_i/o_ proteins and show that pretreatment with PTX (100 ng/ml overnight) completely abrogated the WNT-5A-induced ERK1/2 phosphorylation (P-ERK1/2), indicating that activation of Gα_i/o_ is upstream of the WNT-5A-induced P-ERK1/2 (Figure 3A). On the other hand, PTX did not affect the WNT-5A-induced formation of PS-DVL (Figure 3B) similar to previous findings in SN4741 cells [31] arguing that G protein and DVL signaling could be distinct branches of WNT signaling. These biochemical data are supported by immunocytochemistry experiments showing that cellular P-ERK1/2 immunoreactivity was increased in microglia stimulated with WNT-5A, an effect which was also blocked by PTX pretreatment (Figure 3C).

**Figure 4:**
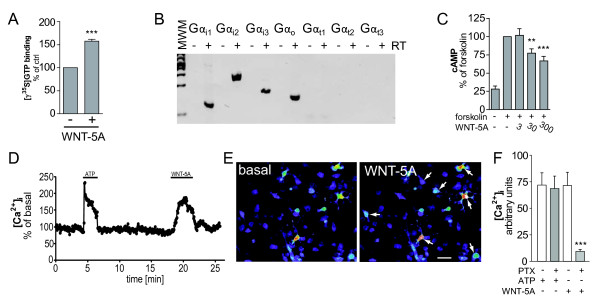
**WNT-5A-induced G protein activation mediates decrease in cAMP and mobilization of [Ca**^**2+**^**]**_**i**_**.** (**A**) Isolated plasma membranes from mouse primary microglia were stimulated with WNT-5A (300 ng/ml) as described in the Methods section to assess activation of heterotrimeric G proteins. Incorporation of [γ-^35^ S]GTP, a measure for activation of G proteins, was quantified by scintillation counting. Data from three independent experiments are shown in the bar graph. ***, *P* < 0.001. Error bars give s.e.m.. (**B**) cDNA from mouse primary microglia was analyzed for expression of PTX-sensitive variants of the G_i/o_ family of Gα subunits by RT-PCR. cDNA synthesis was performed in the absence and presence of RT (+/− RT) to control for the purity of the preparation. (**C**) Detection of intracellular cAMP levels reveals that WNT-5A reduces forskolin-induces cAMP production in a dose-dependent manner (error bars: s.e.m.; n = 3; **, *P* < 0.01; ***, *P* < 0.001.). (**D-F**) Stimulation of Fluo-3-loaded primary microglia with WNT-5A induced fast and transient elevation of [Ca^2+^]_i_. ATP was used as a positive control. The [Ca^2+^]_i_ trace shown in D originates from a single cell. Typically 15% to 30% of the cultured microglia responded to WNT-5A. (**E**) shows a representative view of Fluo-3-loaded cells at baseline and upon WNT-5A (300 ng/ml) exposure. The images are pseudocolored with warm colors presenting high [Ca^2+^]_i_ and cold colors low [Ca^2+^]_i_. Size bar 20 μm. The bar graph summarizes data from five different experiments with ATP and WNT-5A in combination with PTX. Error bar gives s.e.m. n, number; PTX, pertussis toxin; s.e.m., standard error of the mean.

In order to test the assumption that WNT-5A-induced ERK1/2 phosphorylation not only requires active Gα_i/o_ protein but that WNT-5A also directly activates Gα_i/o_, we performed G protein activation assays in membrane preparations from mouse primary microglia stimulated with recombinant WNT-5A (Figure 4A). Activation of heterotrimeric G proteins is measured as an exchange of GDP to GTP, employing a hydrolysis-resistant ^35^ S-labelled GTP analogue, [γ-^35^ S]GTP. Crude [γ-^35^ S]GTP binding assays in mammalian cell membranes are generally suitable to assess GPCR-mediated activation of Gα_i/o_ proteins,due to the advantageous signal to noise ratio in comparison to Gα_s_ or Gα_q/11_ proteins [33]. As shown in Figure 4,A WNT-5A stimulation resulted in GDP/GTP exchange, demonstrating that WNT-5A activates Gα_i/o_ proteins with 157.7 ± 4.3% of control (mean ± s.e.m.) at physiological stochiometry of receptors and G proteins. In order to characterize the expression profile of PTX-sensitive Gα_i/o_ subunits in primary microglia, we performed RT-PCR (Figure 4B), which revealed expression of Gα_i1__i2__i3__o_ but not Gα_t1__2__3_. Consequently, WNT-5A-induced stimulation in microglia can potentially activate Gα_i1-3_ and Gα_o_. Gα_i/o_-coupled GPCRs typically reduce cAMP levels through Gα_i/o_ subunits and induce changes in intracellular calcium ([Ca^2+^_i_) through the release of βγ and the subsequent phospholipase C (PLC)-dependent production of inositoltrisphosphate and calcium release from the endoplasmatic reticulum [22].

WNT-5A was indeed capable of reducing forskolin-induced cAMP levels in a dose-dependent manner, functionally confirming the activation of Gα_i/o_ proteins (Figure 4C). In order to visualize WNT-5A-induced changes in [Ca^2+^_i_ we used Fluo-3-loaded primary microglia cells for live cell imaging. As shown in Figure 4D-F, 300 ng/ml WNT-5A led to a fast and transient increase in [Ca^2+^_i_ in a PTX-sensitive manner reminiscent of previous observations in mammalian cells [34,35].

### **WNT-5A recruits a Gα**_**i**_**, PLC, PKC, MEK1/2 signaling axis to regulate ERK1/2**

**Figure 5:**
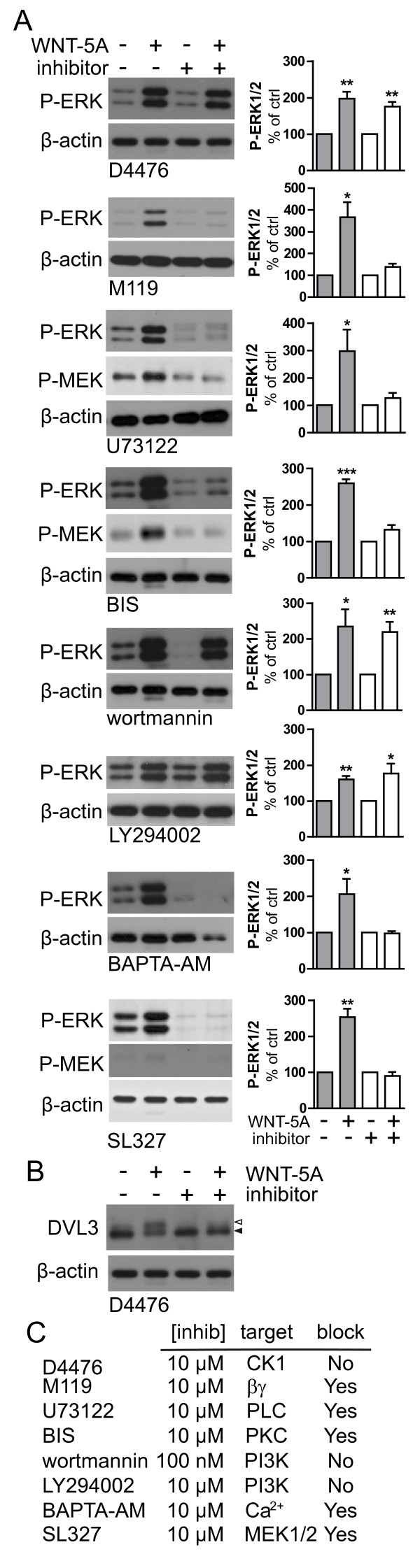
**Mapping of the WNT-5A-induced signaling pathway leading to ERK1/2 phosphorylation in primary microglia.** (**A**) Cells were treated with pharmacological inhibitors 10 minutes prior to WNT-5A challenge (300 ng/ml, 20 minutes). Immunoblotting analysis of P-ERK1/2 and P-MEK1/2 is shown. β-actin serves as loading control. Representative data from at least three experiments are shown and data are quantified in the bar graphs next to each inhibitor (error bars - s.e.m.; *, *P* < 0.05; **, *P* < 0.01; ***, *P* < 0.001). (**B**) Lysates from microglia treated with WNT-5A in the absence or presence of D4479 were analyzed for the formation of PS-DVL3. (**C**) Table summarizes concentrations, targets and effects of the inhibitors used. Pharmacological inhibitors: D4476, CK1 inhibitor; M119, βγ inhibitor; U73122, PLC inhibitor; BIS,(bisindolmaleimide VIII), PKC inhibitor; wortmannin/LY294002, phosphatidylinositol-3'-kinase inhibitor; BAPTA-AM, Ca^2+^-chelator; SL327, MEK1/2 inhibitor. DVL3, disheveled 3; ERK1/2, extracellular signal-regulated kinase 1/2; MEK1/2, MAPK/ERK kinase 1/2; PKC, calcium-dependent protein kinase; PLC, phospholipase C; PS-DVL3, phosphorylated and shifted DVL3; s.e.m, standard error of the mean.

Classical Gα_i/o_-coupled GPCRs can recruit numerous pathways to activate ERK1/2 [22]. Based on our data on G protein activation and calcium mobilization, we hypothesized that WNT-5A could recruit PLC, Ca^2+^ and Ca^2+^-dependent PKC in order to feed into the classical MAPK cascade. To characterize the WNT-5A-induced pathway to ERK1/2 in more detail, we employed a series of pharmacological inhibitors [see also Additional file 1: Table S1]: M119 (inhibits βγ-effector interaction); U73122 (inhibits PLC); bisindolmaleimide VIII (BIS; inhibits PKC); BAPTA-AM (a [Ca^2+^_i_ chelator); wortmannin and LY294002 (inhibit phosphatidylinositol-3'-kinase/PI3K); SL327 (inhibits MEK1/2); and D4476 (casein kinase 1 inhibitor). Preincubation with M119, U73122, BIS, and SL327 but not wortmannin/LY294002 or D4476 completely blocked the WNT-5A-induced ERK1/2 response (Figure 5A), suggesting the involvement of βγ release, PLC, PKC and MEK1/2 but not PI3K and CK1. The activity of D4476 was confirmed by analysis of PS-DVL3 levels: since the formation of PS-DVL3 is dependent on CK1 we could observe a reduced basal shift in DVL3 upon D4476 treatment (Figure 5B) comparable to previous data obtained in other cell types [31,36].

Hence, our data indicate the involvement of βγ subunits released from Gα_i/o_, PLC, PKC, and MEK1/2 in the WNT-5A-induced signaling axis to ERK1/2 and its independence from CK1, PS-DVL3 formation and PI3K. The active involvement of MEK1/2 is corroborated by direct immunoblotting showing that MEK1/2 is phosphorylated upon WNT-5A treatment. In addition, we show that BIS and U73122 block WNT-5A-mediated MEK1/2 phosphorylation, confirming that it is regulated by the same upstream components (Figure 5A).

In summary, this suggests that WNT-5A can activate PS-DVL-dependent pathways, which we have not investigated in more detail in this study. In addition, WNT-5A activates heterotrimeric G protein signaling, which leads to a reduction in cAMP levels and the activation of ERK1/2 through engagement of βγ subunits, PLC, PKC, and MEK1/2. The ERK1/2 cascade is an established proliferative pathway with proinflammatory function in microglia [20,28,29,37]. Thus, based on the signaling profile induced by WNT-5A we suggest that its effects might be of a proliferative and proinflammatory nature in mouse primary microglia.

### **Increased expression of iNOS, COX-2 and TNFα upon WNT-5A exposure**

**Figure 6:**
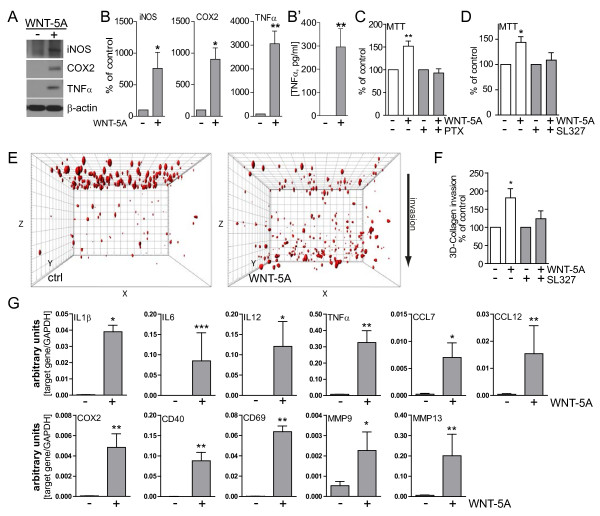
**WNT-5A induces a proinflammatory transformation in mouse microglia.** (**A**, **B**) iNOS, COX-2 and TNFα were detected by immunoblotting in lysates from mouse primary microglia after WNT-5A stimulation (ctrl, 300 ng/ml, 6 hours). (B’) shows TNFα levels in the supernatant of primary microglia under ctrl conditions and upon WNT-5A stimulation (ctrl, 300 ng/ml, 24 hours; n = 4). At least three experiments are summarized in the bar graphs. Data are normalized to ctrl. Error bars give s.e.m. (**C**) Microglial proliferation was assessed by an MTT assay monitoring mitochondrial activity, which is proportional to cell number [see Additional file 1: Figure S5]. Stimulation with WNT-5A (300 ng/ml, 24 hours) increased MTT, which was blocked by PTX (100 ng/ml, overnight) or the MEK1/2 (10 μM) inhibitor, SL327. (**D**). Experiments were done in triplicate and data from three independent experiments are shown. *, *P* < 0.01; ***, *P* < 0.001: Error bars show s.e.m.. (**E**) Cell tracker (red)-stained primary microglia were seeded on top of a collagen matrix in 35 mm glass bottom dishes. One day after ctrl or 300 ng/ml WNT-5A stimulation, invasion was observed by confocal microscopy and Z-stacking using a Zeiss LSM710 microscope and subsequent analysis with the Bitplane Imaris software. The size of the collagen cube shown is 1,000 (Z) x 1,400 (Y) x 1,400 (X) μm. Three invasion experiments in the absence and presence of the MEK1/2 inhibitor SL327 were quantified. Data are presented in a bar graph (**F**). *, *P* < 0.05; error bars show s.e.m.. (**G**) cDNA of ctrl stimulated (−) and WNT-5A stimulated (+) primary microglia was analyzed by QPCR for expression of inflammatory genes. At least three independent experiments are summarized. Gene expression is normalized to the housekeeping gene GAPDH and expressed as arbitrary units (2^-Δct^). *, *P* < 0.05; **, *P* < 0.01. COX-2, cyclooxygenase 2; iNOS, inducible nitric oxide synthase; MTT, 3-(4,5-dimethylthiazol-2-yl)-2,5-diphenyltetrazolium bromide; n, number; s.e.m., standard error of the mean.

To assess the proinflammatory potential of WNT-5A we initially employed immunoblotting to measure regulation of proinflammatory markers, which are generally induced upon activation of microglia: inducible nitric oxide synthase (iNOS), cyclooxygenase 2 (COX-2) and TNFα. iNOS generates the reactive and cell permeable mediator nitric oxide, COX-2 is a crucial player in prostanoid synthesis and TNFα is an important proinflammatory cytokine. Six-hour stimulation with 300 ng/ml WNT-5A increased iNOS, COX-2 and TNFα expression substantially (Figure 6A, B). In order to support the TNFα protein expression data from cell lysates with information on WNT-5A-induced mediator release, supernatants from ctrl- and WNT-5A-stimulated primary microglia were analyzed for TNFα by Mesoscale measurements, indicating an increased release of proinflammatory TNFα (Figure 6B’).

### **Microglia proliferate and invade in response to WNT-5A stimulation**

When microglia cells are activated in vivo, they proliferate and are attracted to the site of injury. The substantial proinflammatory transformation induced by WNT-5A indicated by expression of iNOS, COX-2 and TNFα prompted us to investigate the cellular behavior of WNT-5A-stimulated primary microglia. The WNT-5A-induced activation of ERK1/2, a well-known mediator of cell proliferation, suggested that WNT-5A could also have mitogenic effects. Indeed, stimulation with 300 ng/ml WNT-5A increased mitochondrial activity by143.9 ± 11.2% (mean ± s.e.m.) of control as assessed by an MTT assay (Figure 6C) as well as cell number [see Additional file 1: Figure S5], which in combination is interpreted as an increased proliferation. Employing both PTX and the ethanol-soluble SL327 in combination with the MTT assay, we found support for the hypothesis that the Gα_i/o_-PLC-PKC-MEK1/2-ERK1/2 signaling axis is also important for the WNT-5A-mediated regulation of microglial proliferation (Figure 6C, D). Enhanced capability to invade into the surroundings is a hallmark of microglial activation. Thus, we implemented a three-dimensional collagen invasion assay to assess the invasion of microglia into a collagen matrix. Strikingly, WNT-5A (300 ng/ml; one day) evoked substantial invasion into the collagen matrix as shown in Figure 6E, F. Again, the MEK1/2 inhibitor SL327 blocked the WNT-5A-induced invasive effects completely (Figure 6F).

To further characterize the inflammatory effects of WNT-5A on microglia we charted the inflammatory fingerprint of WNT-5A by gene expression analysis. Quantitative PCR revealed increased expression of the inflammation-related genes IL1β, IL6, IL12, TNFα, CC motif chemokine CCL7, CCL12, clusters of differentiation CD40, CD69, and matrix metalloproteases MMP9 and MMP13 after six hours of treatment with 300 ng/ml WNT-5A (Figure 6G). The observed shift in the expression profile of inflammatory genes with diverse functions clearly corroborates the proinflammatory action of WNT-5A in microglia.

Since we have shown that WNT-5A-induced signaling to ERK1/2 is responsible for the recorded proliferative and invasive effects, we used the MEK1/2 inhibitor SL327 to assess the role of ERK1/2 signaling in the WNT-5A-induced regulation of gene expression. As shown in Table 1, WNT-5A-induced expression of genes for inflammatory markers was affected by SL327 in a bidirectional manner: whereas the increase of MMP9 and MMP13 expression was clearly blocked by MEK1/2 inhibition, the WNT-5A-induced effects on TNFα, CCL7, CCL12, COX-2, and CD40 expression were instead amplified when MEK1/2 was inhibited.

**Table 1:** Inhibition of MEK1/2 by SL327 blocks WNT-5A-induced expression of matrix metalloproteases.

**Gene**	**WNT-5A**	**WNT-5A/SL327**	**Ratio**
IL1β	209.8 ± 78.2	88.1 ± 16.3	0.4
IL6	947.0 ± 554.4	541.0 ± 175.8	0.6
IL12	33622.0 ± 26855.0	31504.0 ± 14202.0	0.9
TNFα	43.0 ± 5.7	366.3 ± 84.5**	8.5
CCL7	29.1 ± 5.1	574.7 ± 348.2*	19.7
_CCL12_	26.7 ± 7.0	354.7 ± 206.3**	12.9
COX-2	93.3 ± 30.4	448.3 ± 169.9*	4.8
CD40	558.0 ± 133.0	1989.0 ± 398.0**	3.6
CD69	456.0 ± 155.0	1723.0 ± 606.0	3.8
MMP9	5.1 ± 1.2	1.0 ± 0.1*	0.2
MMP13	28.3 ± 8.0	6.7 ± 0.9**	0.2

The table summarizes QPCR data obtained from mouse primary microglia stimulated with 300 ng/ml WNT-5A for six hours in the presence and absence of the MEK1/2 inhibitor SL327 (10 μM). Numbers provide the percentage of WNT-5A-induced increase over ctrl or SL327 treatment alone. Results are presented as arbitrary units (fold change of unstimulated control microglia, 2^-ΔΔCt^). The ratio provides a relative measure of decrease (value <1) or increase (>1) of gene expression upon treatment with SL327. Results from at least four independent experiments are shown. Variations are given as s.e.m. *, *P* < 0.05; **, *P* < 0.01.

## **Discussion**

**Figure 7:**
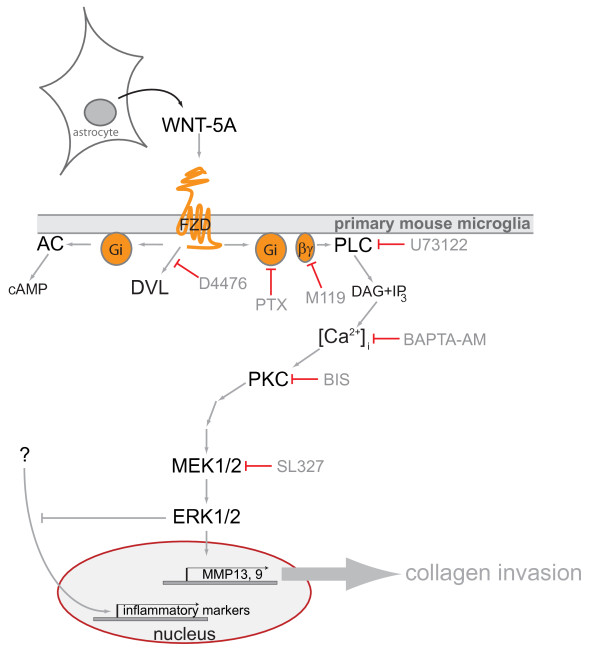
**Schematic overview of the results from the present study indicating WNT-5A secretion from astrocytes and the WNT-5A-induced signaling pathway towards ERK1/2 regulating distinct aspects of the proinflammatory transformation of microglia.** Pharmacological inhibitors used in the study: D4476, pertussis toxin (PTX), M119, BAPTA-AM, U73122, bisindolaleiimide (BIS), SL327. Abbreviations: AC, adenylyl cyclase; cAMP, cyclic AMP; DAG, diacylglycerol; DVL, disheveled; ERK1/2, extracellular signal-regulated kinases 1/2; FZD, Frizzled; IP_3_, inositoltriphosphate; MEK1/2, MAPK/ERK kinase 1/2; MMP, matrix metalloprotease; PLC, phospholipase C; PKC, Ca^2+^-dependent protein kinase; Gi, heterotrimeric G proteins of the G_i/o_ family; WNT, Wingless/int-1.

WNT-5A, which is predominantly expressed in astrocytes in the adult mouse brain, evokes a proinflammatory transformation of microglia characterized by increased expression of cytokines, chemokines and metalloproteases and changes in microglial proliferation and invasiveness. We have characterized underlying signaling pathways, identifying a Gα_i/o_-PLC-PKC-MEK-ERK1/2 signaling axis (see also Figure 7) as necessary for some but not all aspects of the WNT-5A-induced inflammatory transformation. Thus, we report for the first time that WNT-5A-induced signaling through heterotrimeric G proteins and the ERK1/2 cascade is activated in cells with endogenous stoichiometry of WNT receptors and G proteins and that this has physiological relevance for the regulation of microglia in the brain and, thus, for the CNS immune response.

### **WNT-5A-evoked proinflammatory transformation of microglia**

The establishment and maintenance of regional and phenotypic differences in activated microglia are so far only poorly understood. The spectrum of WNT-5A-induced changes suggests that microglia become activated, motile, proliferative, and communicate with their surroundings by means of secreted mediators. IL1β, IL6, IL12 and TNFα act proinflammatory and are known to be secreted by activated microglia in many different conditions, such as neurodegenerative disease, trauma or infection [23,38]. These cytokines serve mainly for communication with surrounding microglia, macroglia and neurons but also infiltrating immune cells. In addition, we have investigated the expression of CC motif chemokines, namely CCL7 and CCL12, which are also known as monocyte chemotactic protein 3 and 5, respectively. These chemokines are important for monocyte and leukocyte recruitment, indicating that WNT-5A-activated microglia could serve to engage peripheral, infiltrating immune cells to support neuroinflammatory processes [39,40]. Also, expression of CD40, a member of the tumor necrosis factor receptor family, and CD69, a C type lectin, serve for communication with astrocytes and peripheral immune cells, such as lymphocytes and natural killer cells [41]. Further, cytokines and chemokines are known to act both in a paracrine and autocrine manner, suggesting that their WNT-5A-induced release could also support communication among microglia to promote and spread the inflammatory response [42]. Additional transmission of inflammatory signals is accomplished by the WNT-5A-induced upregulation of iNOS and COX-2, which both have a crucial role in the inflammatory reaction and produce nitric oxide (NO) and prostaglandins, respectively [43].

### **Regulation of MMPs by WNT-5A**

In addition to increasing NO-, prostaglandin-, cytokine- and chemokine-based intercellular communication, WNT-5A enhanced MMP9 and MMP13 expression, suggesting a role in extracellular matrix remodeling. The function of MMPs in the brain is complex and includes promotion of cell invasion as well as disruption of the blood–brain barrier [44-46]. Thus, increased invasion of microglia and recruitment of peripheral immune cells could be a consequence of MMP secretion from WNT-5A-stimulated microglia. Indeed, this assumption is supported by the WNT-5A-induced microglial invasion in the collagen three dimensional invasion assay (Figure 5E, F). Furthermore, MMPs contribute to the processing, activation and release of non-extracellular matrix proteins, such as growth factors, cytokines, FAS ligand, E-cadherin, integrins, and additional MMPs by a process called ectodomain shedding [47]. MMP13, primarily a collagenase, is known to cleave many substrates [48], among others heparan sulfates. Since MMP13 was shown to promote the release of basic fibroblast growth factor [49] it similarly could release heparan sulfate-bound WNTs thereby amplifying WNT action in the inflammatory microenvironment.

### **WNT-5A-induced ERK1/2 signaling in microglia**

In order to address the role of MEK1/2, the upstream kinase of ERK1/2, for the WNT-5A-induced changes in gene expression, we chose SL327 over more widely used PD98059 or UO126 because SL327 showed a higher solubility in ethanol over dimethyl sulfoxide (DMSO) among those substances. DMSO might interfere with the microglia integrity and activity status in long time stimulation paradigms, such as six or 24 hour, as used in the present study.

Most interestingly, WNT-5A-evoked MMP expression could be blocked by the MEK1/2 inhibitor SL327 (Table 1). On the other hand, WNT-5A-induced TNFα, CCL7, CCL12, COX-2 and CD40 expression was enhanced in the presence of MEK1/2 inhibition. These findings suggest a central regulatory role of the WNT-5A-ERK1/2 pathway that is able to integrate proinflammatory input through a bidirectional regulation of gene expression. It appears that proinflammatory WNT-5A stimulation of primary microglia establishes a distinct reactive phenotype based on an integral inhibitory autoregulation through ERK1/2. ERK1/2 exerts an inhibitory input towards the ERK1/2-independent, WNT-5A-induced upregulation of TNFα, CCL7, CCL12, COX-2 and CD40, unmasked by simultaneous MEK1/2 inhibition (Table 1). Hypothetically, WNT-5A-induced ERK1/2 phosphorylation could also provide negative crosstalk with other proinflammatory agents, thereby limiting the inflammatory transformation or shifting it towards a more invasive and proliferative phenotype. So far we are not able to identify a candidate pathway, where the WNT-5A-ERK1/2 axis could exert negative regulation. The proposed crosstalk of WNT-5A-induced ERK1/2 activation to attenuate other signaling systems could take place on different levels, such as the receptor level, intermediate signaling components, or the transcriptional levels. Further studies will be required to pinpoint candidate links and to map the underlying mechanisms of this regulatory crosstalk.

MAPK signaling in general and ERK1/2 in particular is a well-established pathway underlying CNS inflammation [28,50]. WNT-5A stimulation of primary microglia leads to a Gα_i/o_, PLC, Ca^2+^, PKC, MEK1/2-dependent phosphorylation of ERK1/2, similar to pathways previously described for Gα_i/o_-coupled GPCRs [22]. Accordingly, Gα_i/o_-independent signaling could induce ERK1/2-independent aspects of microglial activation, for example, via formation of PS-DVL. ERK1/2 signaling downstream of GPCRs can almost be seen as a default pathway irrespective of the heterotrimeric G proteins involved and agonist-induced activation of ERK1/2 is localized downstream of many different GPCRs [22].

However, WNT-induced signaling towards ERK1/2 cannot be observed as a general readout, which is confirmed by unpublished observations indicating that WNT-5A does not induce P-ERK1/2 upon 10 minutes of stimulation in human embryonic kidney cells (HEK293), mouse embryonic fibroblasts, a substantia nigra-derived dopaminergic precursor cell line SN4741 or N13 microglia-like cells (not shown).

### **Astrocytes as source of proinflammatory WNT-5A**

Establishment of reactive microglia upon CNS damage precedes the development of astrogliosis, but both processes have overlapping inflammatory functions in CNS pathology [51]. Secreted factors from microglia are known to affect astrocytes and *vice versa*. During midbrain neurogenesis, astrocytes and radial glia were shown to increase the number of Nurr1 dopaminergic precursors through WNT-5A secretion [52], indicating that astroglia are an important source of CNS WNT-5A during development. Our findings now show that cultured astrocytes isolated from newborn mice and astrocytes in intact mouse brain tissue express WNT-5A suggesting a paracrine communication between astrocytes and surrounding cells such as microglia and neurons. Further, we show that mouse primary microglia in comparison to astrocytes express lower levels of WNT-5A mRNA and protein indicating that an autocrine regulation of microglia by WNT-5A could exist but that this might be of minor importance.

So far, the regulation of WNT-5A release from astrocytes to accomplish a proinflammatory transformation of surrounding microglia remains obscure. Since WNT-5A protein levels in astrocytes are prominent also under normal conditions, we suggest that regulation of release mechanisms, possibly depending on transcriptional or functional regulation of Porcupine or WNTless [53] under proinflammatory conditions, could result in increased secretion of WNT-5A.

### **Putative role of WNT-5A-induced proinflammatory transformation in pathophysiology**

Although the role of WNT-5A for brain development has been mapped in some detail, less is known of its involvement in brain disorders in the adult [5,6,54,55]. So far it is not known if levels of WNT-5A or its receptors are dynamically regulated under pathophysiological conditions, such as neurodegenerative disease, infection, trauma, hypoxia or other inflammatory conditions. One exception is brain tumors and metastases, which have been associated with overexpression of WNT-5A [56,57]. Brain tumors also increase the astrocyte- and microglia-dependent immune response and the presence of activated tumor-associated microglia was reported a long time ago [58]. In fact, recent data show that WNTs, and especially WNT-5A, signaling in microglia/macrophages, can promote invasiveness of breast cancer cells and especially their metastasis in the brain [59-61]. Most importantly, our study shows that microglia cells are WNT-receiving cells, which interpret WNT-5A stimulation with a strong proinflammatory output.

## **Conclusions**

In summary, we identify WNT-5A originating from astrocytes as a proinflammatory regulator of microglia, capable of increasing their number and invasiveness, thereby supporting basic immune function in the CNS. Further, we show that distinct subsets of WNT-5A-induced effects on microglial function are mediated through the ERK1/2 cascade. Thus, we conclude that WNT-5A has an important role to define a distinct proinflammatory phenotype of microglia in pathophysiological conditions associated with neuroinflammation. Future studies will reveal the putative crosstalk between G protein-dependent and -independent WNT-5A signaling in microglia and its role for the establishment and maintenance of a reactive microglia phenotype under different neuroinflammatory conditions.

## **Abbreviations**

ANOVA, analysis of variance; BAPTA-AM, 1,2-bis(2-aminophenoxy)ethane-N,N,N,N-tetraacetic acid tetrakis (acetoxymethylester); BIS, bisindolmaleimide VIII; CCL, chemokine (CC-motif) ligand; CK, casein kinase; CNS, central nervous system; COX-2, cyclooxygenase 2; D4476, 4-(4-(2,3-dihydrobenzo[1,4]dioxin-6-yl)-5-pyridin-2-yl-1 H-imidazol-2-yl)benzamide; (D)MEM, (Dulbecco’s) modified Eagle’s medium; DVL, disheveled; ERK1/2, extracellular signal-regulated kinase 1/2; ELISA, enzme-linked immunosorbent assay; FBS, fetal bovine serum; FZD, Frizzled; GFAP, glial fibrillary acidid protein; GPCR, G protein-coupled receptor; IBA1, ionized calcium-binding adaptor protein 1; IL, interleukin; iNOS, inducible nitric oxide synthase; LY294002, 2-(4-morpholinyl)-8-phenyl-1(4 H)-benzopyran-4-one hydrochloride; MAPK, mitogen-activated protein kinase; MEK, MAPK/ERK1/2 kinase; MMP, matrix metalloprotease; MTT, 3-(4,5-dimethylthiazol-2-yl)-2,5-diphenyltetrazolium bromide; PBS, phosphate buffered saline; PCR, polymerase chain reaction; PI3K, phosphatidylinositol-3'-kinase; PKC, Ca2+-dependent protein kinase; PLC, phospholipase C; PS-DVL, phosphorylated and shifted DVL; PTX, pertussis toxin; QPCR, quantitative reverse transcriptase PCR; s.e.m., standard error of the mean; SFRP, secreted Frizzled-related protein; SL327, α-[amino-(4-aminophenylthio)methylene)-2-(trifluoromethyl)phenylacetonitrile; TNFα, tumor necrosis factor α; U73122, 1-[6-[((17β)-3-methoxyestra-1,3,5[10]-trien-17-yl)amino]hexyl]-1 H-pyrrole-2,5-dione; WNT, acronym composed of Wingless/Int-1.

## **Competing interests**

The authors declare that they have no competing interests.

## **Authors’ contributions**

CH, JPD, MBCK, EL, JBO and JCV performed experiments and collected data. CH, JPD, MBCK, EL, JBO, JCV and GS analyzed data and prepared the figures. GS, CH, EA and JPD planned the experiments. CH, GS, JPD, MBCK wrote and EA, JCV revised the manuscript. All authors agreed to the final version of the manuscript.

## Supplementary Material

Additional file 1 **Figure S1:** FZD expression in mouse primary microglia. The bar graph depicts expression levels of FZD1-10 in mouse primary microglia measured by quantitative PCR. Error bars provide standard SEM. N = . Probe efficiency for GAPDH and FZD probes was determined using the CT slope method over six cDNA dilutions. Slope/R2 in %: GAPDH: - 3.3745/99.96%; FZD1: -3.5317/99.88%; FZD2: -3.5317/99.88%; FZD3: -3.2678/99.82%; FZD4: -.4883/99.64%; FZD5: -3.4356/97.65%; FZD6: -3.2966/99.65%; FZD7: -3.6285/99.56; FZD8: -3.3238/99.98%; FZD9: -3.3352/99.99%; FZD10: -3.2384/99.40%. Thus, direct comparison and quantitative statements about relative FZD expression levels are justified. We have previously reported that expression levels of non-GPCR coreceptors for WNTs, such as ROR1/2 and RYK, are low in both microglia cell lines and primary microglia (Halleskog *et al.*, 2011; Kilander *et al.*, 2011). **Figure S2:** In order to confirm the specificity of the WNT-5A-induced P-ERK1/2 response and to exclude unspecific responses of microglia to putative contaminants in the WNT preparation, we employed recombinant soluble FZD-related protein 1 (SFRP1). SFRP1 binds WNTs thereby preventing their interaction with the receptor (Kawano and Kypta, 2003; Kilander *et al.*, 2011). Stimulation of primary microglia with WNT-5A (300 ng/ml) in the presence of SFRP1 (10 μg/μl) did not result in increased P-ERK1/2 arguing that SFRP1 sequesters WNT-5A selectively preventing its effects. N = 3. **Figure S3:** WNT-5A-induced β-catenin-independent WNT signaling. (**A**) Immunoblotting analysis of WNT-3A and WNT-5A stimulated cell lysates from mouse primary microglia cells reveals a WNT-5A-induced formation of PS-DVL3 but a lack of β-catenin stabilization and LRP6 phosphorylation. WNT-5A induced the dynamic formation of PS-DVL3 in a time- (*B*) and dose-dependent (**C**) manner. β-actin was employed as a loading control. N ≥3. **Figure S4:** Mouse primary microglia cultures seeded on gelatin-coated glass cover slips were routinely stained with indirect immunocytochemistry for the microglia marker CD11b and glial fibrillary acidic protein (GFAP). Counterstaining for nuclear DNA was done with DAPI. Cellular morphology is shown in the bright field image. Microglia culture contained >95% CD11b+/GFAP- cells with microglial morphology. GFAP + astrocytes were only detected occasionally. Size bar: 50 μm. **Figure S5:** Mouse primary microglia cultures were stimulated with ctrl or 300 ng/ml WNT-5A for 24 hours. Cells were then manually counted in a Bürker chamber and data (normalized to control) summarized in a bar graph. WNT-5A increased cell number to 129.2 ± 6.5 (mean ± s.e.m.). Variation is shown as s.e.m. (n = 4). **, *P* <0.01. **Table S1:** Pharmacological inhibitors used in this study [62,63]. Click here for file
