# Supplementary material for: Heterotrimeric G protein-dependent WNT-5A signaling to ERK1/2 mediates distinct aspects of microglia proinflammatory transformation
Source: J Neuroinflammation. 2012 May 30;9:111. doi: 10.1186/1742-2094-9-111 (PMC3458933; doi:10.1186/1742-2094-9-111)
Supplement: Additional file 1 — Figure S1: FZD expression in mouse primary microglia. The bar graph depicts expression levels of FZD1-10 in mouse primary microglia measured by quantitative PCR. Error bars provide standard SEM. N = . Probe efficiency for GAPDH and FZD probes was determined using the CT slope method over six cDNA dilutions. Slope/R2 in %: GAPDH: - 3.3745/99.96%; FZD1: -3.5317/99.88%; FZD2: -3.5317/99.88%; FZD3: -3.2678/99.82%; FZD4: -.4883/99.64%; FZD5: -3.4356/97.65%; FZD6: -3.2966/99.65%; FZD7: -3.6285/99.56; FZD8: -3.3238/99.98%; FZD9: -3.3352/99.99%; FZD10: -3.2384/99.40%. Thus, direct comparison and quantitative statements about relative FZD expression levels are justified. We have previously reported that expression levels of non-GPCR coreceptors for WNTs, such as ROR1/2 and RYK, are low in both microglia cell lines and primary microglia (Halleskog et al., 2011; Kilander et al., 2011). Figure S2: In order to confirm the specificity of the WNT-5A-induced P-ERK1/2 response and to exclude unspecific responses of microglia to putative contaminants in the WNT preparation, we employed recombinant soluble FZD-related protein 1 (SFRP1). SFRP1 binds WNTs thereby preventing their interaction with the receptor (Kawano and Kypta, 2003; Kilander et al., 2011). Stimulation of primary microglia with WNT-5A (300 ng/ml) in the presence of SFRP1 (10 μg/μl) did not result in increased P-ERK1/2 arguing that SFRP1 sequesters WNT-5A selectively preventing its effects. N = 3. Figure S3: WNT-5A-induced β-catenin-independent WNT signaling. (A) Immunoblotting analysis of WNT-3A and WNT-5A stimulated cell lysates from mouse primary microglia cells reveals a WNT-5A-induced formation of PS-DVL3 but a lack of β-catenin stabilization and LRP6 phosphorylation. WNT-5A induced the dynamic formation of PS-DVL3 in a time- (B) and dose-dependent (C) manner. β-actin was employed as a loading control. N ≥3. Figure S4: Mouse primary microglia cultures seeded on gelatin-coated glass cover slips were routinely stained [file 1742-2094-9-111-S1.pdf]

Heterotrimeric G protein-dependent WNT-5A signaling to ERK1/2 mediates distinct aspects of microglia proinflammatory transformation

Proinflammatory WNT-5A signaling in microglia

Carina Halleskog[1], Jacomijn Petronella Dijksterhuis[1], Michaela Brita Christina Kilander[1], Javier Becerril Ortega[1], J Carlos Villaescusa[2], Eva Lindgren[1], Ernest Arenas[2], Gunnar Schulte[1]

[1]Karolinska Institutet, Dept. Physiology & Pharmacology, Sec Receptor Biology & Signaling, Nanna Svartz väg 2, S-17177 Stockholm, Sweden

[2] Karolinska Institutet, Dept. Medical Biochemistry & Biophysics, Sec. Molecular Neurobiology, Retzius väg 8, S-17177 Stockholm, Sweden

corresponding author:

Gunnar Schulte, PhD
Karolinska Institutet
Dept. Physiology & Pharmacology
Sec. Receptor Biology & Signaling
Nanna Svartz väg 2
S-171 77 Stocklholm
e-mail: gunnar.schulte@ki.se
phone: +46-852487933
fax: +468341280

Fig. S1: FZD expression in mouse primary microglia. The bar graph depicts expression levels of $FZD_{1\text{-}10}$ in mouse primary microglia measured by quantitative PCR. Error bars provide standard SEM. N=3. Probe efficiency for GAPDH and $FZD_{3, 5, 7, 8}$ probes was determined using the C_T slope method over 6 cDNA dilutions. Slope/R^2 in %: GAPDH: -3.3745/99.96%; FZD_1: -3.5317/99.88%; FZD_2: -3.5317/99.88%; FZD_3: -3.2678/99.82%; FZD_4: -3.4883/99.64%; FZD_5: -3.4356/97.65%; FZD_6: -3.2966/99.65%; FZD_7: -3.6285/99.56; FZD_8: -3.3238/99.98%; FZD_9: -3.3352/99.99%; FZD_{10}: -3.2384/99.40%. Thus, direct comparison and quantitative statements about relative FZD expression levels are justified. We have previously reported that expression levels of non-GPCR coreceptors for WNTs, such as ROR1/2 and RYK, is low in both microglia cell lines and primary microglia (Halleskog et al., 2011; Kilander et al., 2011).

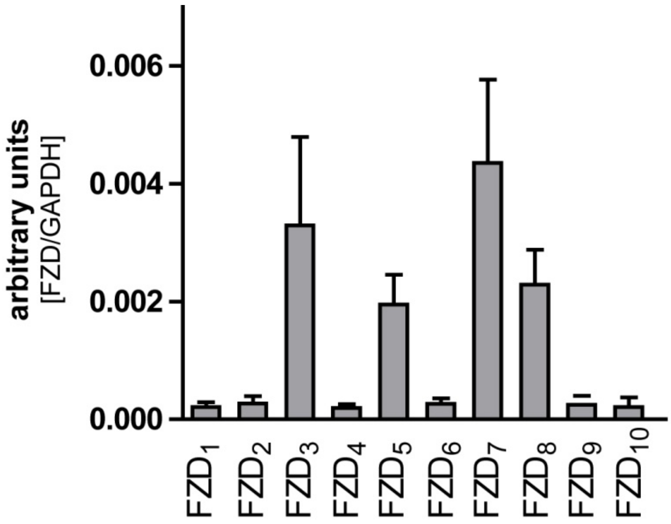

Fig. S2: In order to confirm the specificity of the WNT-5A-induced P-ERK1/2 response and to exclude unspecific responses of microglia to putative contaminants in the WNT preparation, we employed recombinant soluble FZD-related protein 1 (SFRP1). SFRP1 binds WNTs thereby preventing their interaction with the receptor (Kawano and Kypta, 2003; Kilander et al., 2011). Stimulation of primary microglia with WNT-5A (300 ng/ml) in the presence of SFRP1 (10 µg/µl) did not result in increased P-ERK1/2 arguing that SFRP1 sequesters WNT-5A selectively preventing its effects. N=3.

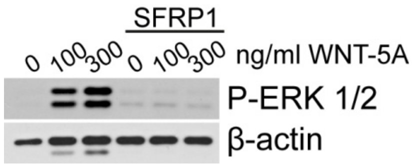

Fig. S3: WNT-5A-induced β-catenin-independent WNT signaling. (A) Immunoblotting analysis of WNT-3A and WNT-5A stimulated cell lysates from mouse primary microglia cells reveals a WNT-5A-induced formation of PS-DVL3 but a lack of β-catenin stabilization and LRP6 phosphorylation. WNT-5A induced the dynamic formation of PS-DVL3 in a time- (B) and dose-dependent (C) manner. β-actin was employed as a loading control. N≥3.

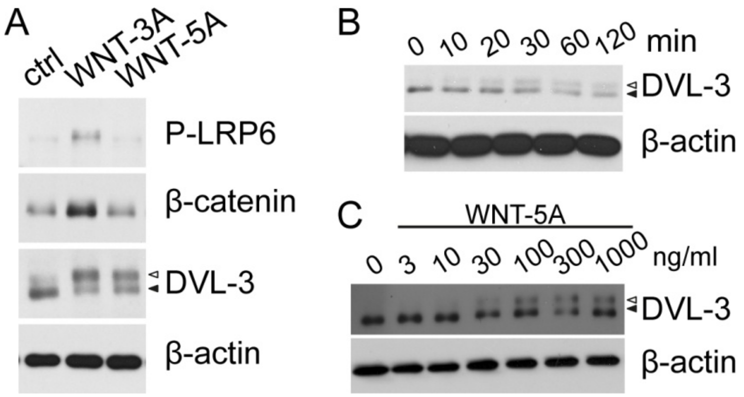

Fig. S4: Mouse primary microglia cultures seeded on gelatine-coated glass cover slips were routinely stained with indirect immunocytochemistry for the microglia marker CD11b and glial fibrillary acidic protein (GFAP). Counterstaining for nuclear DNA was done with DAPI. Cellular morphology is shown in the bright field image. Microglia culture contained >95% CD11b^{+}/GFAP^{-} cells with microglial morphology. GFAP^{+} astrocytes were only detected occasionally. Size bar: 50 µm.

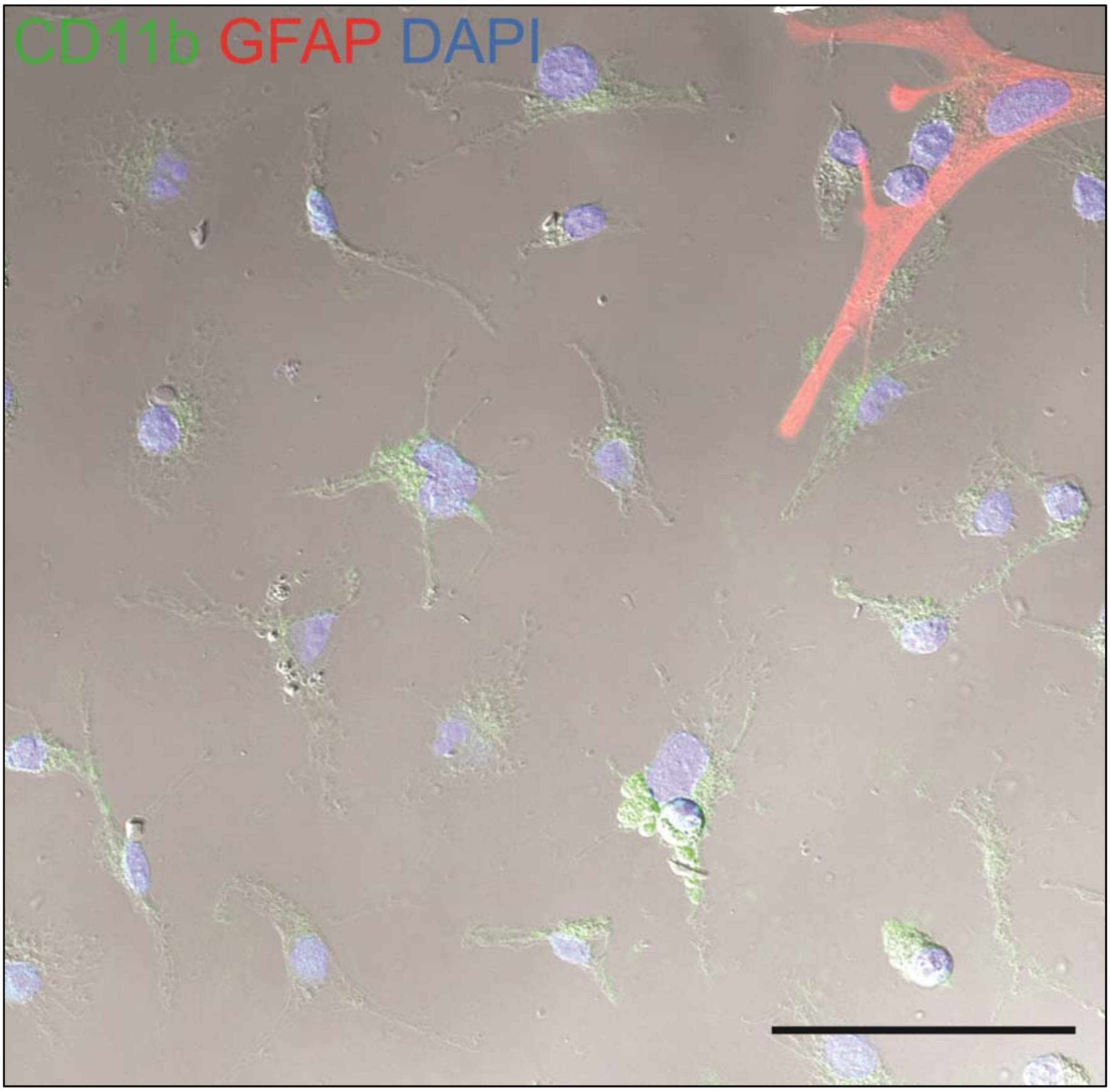

Fig. S5: Mouse primary microglia cultures were stimulated with ctrl or 300 ng/ml WNT-5A for 24 h. Cells were then manually counted in a Bürker chamber and data (normalized to control) summarized in a bar graph. WNT-5A increased cell number to 129,2 ± 6,5 (mean ± s.e.m.). Variation is shown as s.e.m. (n=4). **, *P*<0.01.

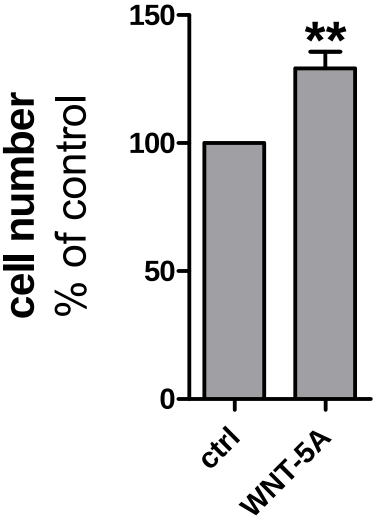

Table S1: Pharmacological Inhibitors used in this study:

Inhibitor	Target	[inhibitor]	Name
PTX	$G\alpha_{i/o}$	100 ng/ml	pertussis toxin
M119	$\beta\gamma$	10 µM	NSC119910; 2-(4,5,6-Trihydroxy-3-oxo-3H-xanthen-9-yl)-cyclohexane-1-carboxylic acid
D4476	Casein kinase 1	10 µM	4-(4-(2,3-dihydrobenzo[1,4]dioxin-6-yl)-5-pyridin-2-yl-1H-imidazol-2-yl)benzamide
U73122	Phospholipase C	10 µM	1-[6-[((17β)-3-Methoxyestra-1,3,5[10]-trien-17-yl)amino]hexyl]-1H-pyrrole-2,5-dione
BAPTA-AM	$[Ca^{2+}]_i$	10 µM	1,2-bis(2-Aminophenoxy)ethane-*N*,*N*,*N*,*N*-tetraacetic acid tetrakis (acetoxymethylester)
BIS	Calcium-dependent protein kinase		Bisindolmaleiimide
wortmannin	Phosphatidylinositol-3'-kinase	100 nM	wortmannin
LY294002	Phosphatidylinositol-3'-kinase	10 µM	2-(4-morpholinyl)-8-phenyl-1(4H)-benzopyran-4-one hydrochloride
SL327	MAPK/ERK1/2 kinase	10 µM	α-[Amino-(4-aminophenylthio)methylene)-2-(trifluoromethyl)phenylacetonitrile

References

Halleskog, C., Mulder, J., Dahlström, J., Mackie, K., Hortobágyi, T., Tanila, H., Kumar Puli, L., Färber, K., Harkany, T. and Schulte, G. (2011). WNT signaling in activated microglia is pro-inflammatory. Glia 59(1), 119-131.

Kawano, Y. and Kypta, R. (2003). Secreted antagonists of the Wnt signalling pathway. J. Cell Sci. 116(Pt 13), 2627-2634.

Kilander, M., Halleskog, C. and Schulte, G. (2011). Purified WNTs differentially activate beta-catenin-dependent and -independent pathways in mouse microglia-like cells. Acta Physiologica 203(3), 363-72.
